# Longitudinal associations between the COVID-19 pandemic and sleep characteristics in children and parents

**DOI:** 10.3389/fpubh.2025.1604195

**Published:** 2025-09-25

**Authors:** Deborah Wernecke, Raphael S. Peter, Stefanie Braig, Maricruz Zarco, Jon Genuneit, Dietrich Rothenbacher

**Affiliations:** ^1^Institute of Epidemiology and Medical Biometry, Ulm University, Ulm, Germany; ^2^German Center for Child and Adolescent Health (DZKJ), Partner Site Ulm, Ulm, Germany; ^3^Pediatric Epidemiology, Department of Pediatrics, Medical Faculty, Leipzig University, Leipzig, Germany; ^4^German Center for Child and Adolescent Health (DZKJ), Partner Site Leipzig, Leipzig, Germany

**Keywords:** Children's Sleep Habits Questionnaire (CSHQ), COVID-19 pandemic, school-aged children, longitudinal data, families, Pittsburgh Sleep Quality Index (PSQI), sleep quality

## Abstract

**Introduction:**

The COVID-19 pandemic and associated preventive measures influenced family health and behavior, leading to diverse effects on sleep.

**Methods:**

This study examined sleep characteristics before, during and after the COVID-19 pandemic in children (*n* = 558), mothers (*n* = 465), and fathers (*n* = 318) in Germany, using data from the prospective Ulm SPATZ Health Study. We compared the period from April 2017 to May 2023 (children aged 5–10 years) with the period from 15 March 2020 to 3 April 2022, defined as “COVID-19 pandemic.” Sleep quality was measured using the Children's Sleep Habits Questionnaire (CSHQ) and the Pittsburgh Sleep Quality Index (PSQI), among children and parents, respectively. Multivariable mixed models were used to assess the associations of the COVID-19 pandemic compared to before and after the pandemic with sleep characteristics among boys, girls, mothers, and fathers, separately.

**Results:**

Child and parent sleep quality showed a weak correlation among 6-year-old boys and their mothers. A moderate correlation was observed between mothers' mental health and boys' sleep quality at ages 5, 6, and 9. Parents' mental health showed a moderate correlation with their sleep quality. Multivariable mixed models revealed better sleep quality (lower CSHQ total scores) among boys during the COVID-19 pandemic compared to before and after. Daytime sleepiness decreased among boys and girls, while no significant changes were found for sleep duration and sleep latency. Among mothers, sleep duration increased on free-days, while fathers experienced increased sleep duration on work-days, along with increased sleep efficiency.

**Discussion:**

This study showed that family sleep quality (indexed with the CSHQ and PSQI) did not decrease during the COVID-19 pandemic compared to sleep quality measured at one (parents) or two (children) annual time points before the pandemic and up to one after it. Instead, parental sleep duration increased, children experienced reduced daytime sleepiness, and boys exhibited improved sleep quality. To effectively optimize public health during a pandemic, findings on sleep quality should be interpreted in conjunction with findings on mental health, given their interrelated nature, as also indicated by our study.

## Introduction

The COVID-19 pandemic, along with the legally mandated preventive measures implemented to mitigate its spread, have affected the health and behavior of children and parents. Sleep is a critical aspect of health (1), and the pandemic has affected sleep characteristics in several ways (2–4). While sleep duration may increase (2, 4–11), it is important to note that poorer mental health (12–14), anxiety (15, 16), increased screen-time (2, 17–19), and altered and irregular sleep patterns (2, 8, 9, 20, 21) during a pandemic may negatively impact other important aspects of sleep (9, 22, 23), such as sleep disturbances or sleep latency. The underlying mechanism and family-specific pathways are described by Peltz et al. (24), Ballesio et al. (25) and Zhan et al. (26). These factors may contribute to the overall decline in subjective sleep quality observed among children, adolescents (2, 9, 27–30), and parents (30–32) during the pandemic. Studies investigating sleep characteristics within families and their association with the COVID-19 pandemic remain limited and have been rarely analyzed to date.

The current study aimed to investigate associations of the COVID-19 pandemic (compared to pre- and and post-pandemic periods) with sleep characteristics of children and parents using the Ulm SPATZ Health Study. This longitudinal birth cohort study has conducted annual follow-ups with children and parents since 2012, providing data spanning the pre-pandemic, pandemic, and post-pandemic.

## Methods

### Study participants

The current study was based on the Ulm SPATZ Health study, a population-based longitudinal birth cohort study conducted in Ulm, southern Germany. Participants were recruited at the Department of Gynecology and Obstetrics, University Hospital Ulm (at that time the only maternity hospital in Ulm). Mothers who had insufficient German language skills, had an outpatient childbirth, were under the age of 18, had a postpartum transfer to the intensive care unit, or experienced a stillbirth were excluded from participating in the study. A complete description of the Ulm SPATZ Health study can be found elsewhere (33). Briefly, 1,006 newborns from 970 mothers were recruited while hospitalized following delivery. Baseline data were collected between April 2012 and May 2013 (overall response rate, 49%). Children and parents have been followed up annually; resulting in the availability of pre-pandemic, COVID-19 pandemic and post-pandemic data.

In the present study, of the 1,006 newborns, a children's subgroup of 558 (55.5% of baseline), was included, for whom at least one questionnaire was completed at ages 5, 6, 7, 8, 9, or 10 years and for whom outcome data were available. The subgroups of mothers and fathers consist of participants who completed at least one questionnaire when the child was aged 6, 7, 8, or 9 years, and for whom outcome data were available. These subgroups included 465 mothers (45.1% of baseline) and 318 fathers (45.0% of baseline). Ethical approval was obtained from the Ethics Committee of the University of Ulm (no. 311/11).

### COVID-19 pandemic

For this analysis, we examined the period from April 2017 to May 2023 (child ages 5-10 years), with the timeframe from March 15, 2020 to April 3, 2022, defined as “COVID-19 pandemic.” Since the questionnaires used assess sleep quality retrospectively over the “last 30 days,” Pittsburgh Sleep Quality Index (PSQI) or “last months,” Children's Sleep Habits Questionnaire (CSHQ), questionnaires completed up to April 15, 2022, were included in the “COVID-19 pandemic” category. Questionnaires completed outside this period were categorized as “before and after the pandemic.” This classification was used to create a dichotomous exposure variable.

### Measurement of sleep characteristics and mental health

#### Sleep characteristics

Child sleep characteristics at ages 5, 6, 7, 8, 9, and 10 years were the primary outcome variables of interest. Sleep quality in children was evaluated using the validated German version of the Children's Sleep Habits Questionnaire (CSHQ) (34), which was completed annually by the parents, mostly the mothers. The CSHQ (35) includes 48 items, of which 33 are categorized into eight different subscales, namely (1) bedtime resistance, (2) sleep latency/sleep onset delay, (3) sleep duration, (4) sleep anxiety, (5) night wakings, (6) parasomnias, (7) sleep-disordered breathing, and (8) daytime sleepiness. Each subscale comprises multiple items, with higher scores indicating poorer sleep quality. The total CSHQ score was calculated by summing all subscale scores, providing a comprehensive measure of overall sleep quality, by incorporating key attributes of sleep quality in children (36). The total CSHQ score can range from 33–99, as each item is reported on a three-point scale. Internal consistency of the validated German version of the CSHQ, measured by Cronbach's alpha, is α = 0.68 (34).

Parent sleep characteristics, assessed when their child was aged 6, 7, 8, and 9 years, were the primary outcome variables of interest. Parent sleep quality was evaluated with the validated German version of the Pittsburgh Sleep Quality Index (PSQI) (37, 38), for work-days and free-days separately. This distinction was based on findings from Pilz et al. (39) and Reis et al. (40) suggesting that the original PSQI predominantly reflects sleep quality on work-days. The PSQI includes seven subscales capturing various characteristics of sleep quality: (1) subjective sleep quality (2) sleep latency, (3) sleep duration, (4) sleep efficiency, (5) sleep disturbance, (6) use of sleep medication, (7) daytime dysfunction. The total PSQI score is calculated by summing the 19 item scores, resulting in an overall sleep quality score ranging from 0 to 21. Internal consistency of the German version of the PSQI, measured by Cronbach's alpha, is α = 0.85 (38).

We also assessed sleep duration, defined as the time between 'falling asleep' and 'waking up', and sleep latency, defined as the time needed to fall asleep, analyzed separately for work-days (weekdays for children) and free-days for both children and parents. Additionally, bedtime was calculated for parents and defined as the actual time spent in bed, analyzed separately for work-days and free-days.

#### Mental health

The German version of the HADS was used to index maternal and paternal mental health at child ages 5, 6, and 9 years. The HADS questionnaire is a validated 14-item screening measure with two subscales that assess symptoms of anxiety (7 items) and depression (7 items) in the last week prior to assessment (41, 42). The questionnaire has also been validated in the German language (42, 43). The possible HADS score ranges from 0–42 (total score) (42). Internal consistencies, measured by Cronbach's alpha, is α = 0.72, and α = 0.82 for the subscales HADS-Anxiety, and HADS-Depression, respectively (44). We chose to use the total HADS score rather than analyzing the anxiety and depression subscales separately, as our primary interest was in the overall psychological distress experienced by participants.

### Confounding variables

Information on parental sociodemographic variables were collected at childbirth through self-reported questionnaires. Child gender assigned at birth (male or female) was used for stratification of the analysis of children.

Confounders were selected based on a review of relevant literature (2, 4, 7, 12, 13, 17, 21, 24, 27, 45–51). For the model between the COVID-19 pandemic and child sleep characteristics; child age at time of the assessment, child age at school entry, maternal educational attainment (<12 or ≥12 years of school education), maternal nationality (German or non-German), and maternal age at childbirth were included to control for confounding. Additionally, the final model was adjusted for maternal mental health, measured using the Hospital Anxiety and Depression Scale (HADS) score when the child was 5 years old (the first longitudinal data point included in the analysis). This adjustment was made to evaluate the association between the COVID-19 pandemic and child sleep characteristics independently of maternal pre-pandemic mental health (we adjusted for maternal pre-pandemic mental health rather than paternal, due to greater completeness of maternal data). “Mental health during the pandemic” was considered a potential mediator, and therefore no adjustment was made for this variable.

For the model between the COVID-19 pandemic and maternal and paternal sleep quality; maternal and paternal age at childbirth, maternal and paternal educational attainment (<12 or ≥12 years of school education), maternal and paternal nationality (German or non-German), and child age at time of the outcome assessment were included to control for confounding. Additionally, the final model was adjusted for maternal and paternal mental health using the HADS score when the child was 6 years old (the first longitudinal data point included in the analysis). This adjustment was made to evaluate the association between the COVID-19 pandemic and maternal and paternal sleep characteristics independently of maternal and paternal pre-pandemic mental health.

### Statistical analysis

Descriptive values of sleep characteristics were analyzed longitudinally and reported by means and standard deviation or n and percentages. Pearson correlation coefficients were calculated to evaluate the strength and direction of linear relationships between concurrent parent and child sleep quality when the child was aged 6 and 8 years (i.e., before and during the COVID-19 pandemic). Additionally, Pearson correlation coefficients were calculated between concurrent parental mental health and child sleep quality when the child was aged 5, 6, and 9 years. Data on parental mental health were unavailable for child ages 7 and 8 years; therefore, correlations for these time points could not be calculated. Lastly, Pearson correlation coefficients were calculated to evaluate the strength and direction of linear relationships between concurrent parental mental health and parental sleep quality when the child was aged 6 and 9 years. Correlations were not calculated for child age 5 years, as parents sleep quality was not assessed separately for work-days and free-days at that time point. These correlations were examined to inform potential associations and underlying patterns, providing insights into the relationship between child sleep quality, parent sleep quality and parental mental health.

We fitted multivariable linear mixed-effects models, assuming an unstructured covariance matrix, to estimate differences in means associated with the time during the COVID-19 pandemic vs. before and after the COVID-19 pandemic. The intercept represents the individual score at baseline (random effect). The models were analyzed separately for boys, girls, mothers, and fathers in order to account for potential effect modification of the COVID-19 pandemic with gender. Based on the availability of sleep data, the mixed models incorporated up to six time points for children (ages 5, 6, 7, 8, 9, and 10 years) and up to four time points for parents (ages 6, 7, 8, and 9 years of the child). Participants were included in the mixed-effects model if they had at least one measurement of the outcome variable available and complete data for all the covariates. Consequently, those with missing data for either the outcome and/or the covariates were excluded from the analysis (listwise deletion). The analysis was performed using SAS^®^ 9.4 (The SAS Institute, Cary, NC, USA).

## Results

### Descriptive results

We analyzed data from 558 children (278 boys, 280 girls), 465 mothers, and 318 fathers with available follow-up data on sleep characteristics. Maternal and paternal age at childbirth did not differ substantially between the baseline sample (*n* = 970 families) and the analysis sample. However, the analysis sample included a slightly higher proportion of mothers with German nationality and a higher educational level compared to the baseline sample (Table 1). The mean age of the children in the analysis sample at school entry was 6.5 years (SD 0.28; rang: 5.97–6.96 years).

**Table 1:** Baseline characteristics of parental data included in the statistical analysis (*n* = 465 mothers, *n* = 318 fathers) vs. SPATZ cohort baseline (*n* = 970 mothers, and *n* = 707 fathers).

	**Baseline SPATZ study**		**Analysis of child sleep; CSHQ available (*****n*** = **558)**		**Analysis of maternal sleep; PSQI available (*****n*** = **465)**		**Analysis of paternal sleep; PSQI available (*****n*** = **318)**	
	***n***	**Mean (SD) or** ***N*** **(%)**	***n***	**Mean (SD) or** ***N*** **(%)**	***n***	**Mean (SD) or N (%)**	***n***	**Mean (SD) or** ***N*** **(%)**
Maternal age at birth	969	32.7 (4.8)	558	33.4 (4.4)	465	33.3 (4.4)	318	33.4 (4.4)
Missing	1	–	–	–	–	–	–	–
Paternal age at birth	603	36.1 (6.1)	412	36.4 (6.3)	350	36.1 (6.2)	318	36.2 (6.1)
Missing	104	–	146	–	115	–	–	–
**Maternal nationality**								
German	962	857 (89.1)	558	518 (92.8)	465	432 (92.9)	318	299 (94.0)
Non-German		105 (10.9)		40 (7.2)		33 (7.1)		19 (6.0)
Missing	8	–	–	–	–	–	–	–
**Paternal nationality**								
German	576	527 (91.5)	399	362 (90.7)	339	308 (90.9)	318	290 (91.2)
Non-German		49 (8.5)		37 (9.3)		31 (9.1)		28 (8.8)
Missing	131	–	159	–	126	–	–	–
**Maternal educational attainment**								
≥12 years	951	561 (59.0)	558	385 (69.9)	465	326 (70.1)	315	233 (74.0)
10–11 years		297 (31.2)		148 (26.5)		120 (25.8)		74 (23.5)
≤ 9 years		93 (9.8)		25 (4.5)		19 (4.1)		8 (2.5)
Missing	19	–	–	–	–	–	3	–
**Paternal educational attainment**								
≥12 years	575	395 (68.7)	400	287 (71.8)	340	242 (71.2)	318	230 (72.3)
<12 years		180 (31.3)		113 (28.3)		98 (28.8)		88 (27.7)
Missing	395	–	158	–	125	–	–	–

CSHQ, Children's Sleep Habits Questionnaire; PSQI, Pittsburgh Sleep Quality Index.

Table 2 presents descriptive data on sleep characteristics among children, mothers and fathers. The results indicate that girls generally exhibited better sleep quality (CSHQ) than boys at all time points, except during the early phase of the COVID-19 pandemic. Mothers reported slightly poorer sleep quality (PSQI) compared to fathers across the study period. Descriptive data suggest differences in sleep duration between boys and girls on week-days and free-days, with girls tending to sleep slightly more on free-days and slightly less on week-days compared to boys. Before the COVID-19 pandemic, when children were 6 years old, 71% of mothers and 73% of fathers reported good sleep quality (PSQI ≤ 5). During the COVID-19 pandemic, when children were 8 years old, these proportions increased slightly to 73% for mothers and 75% for fathers. However, it is important to note that there was a loss to follow up of 66 mothers and 59 fathers between these two time-points. Regarding mental health, 28% of mothers and 10% of fathers reported significant anxiety symptoms (HADS score ≥8) before the COVID-19 pandemic (child age 6 years), while 16% of mothers and 13% of fathers reported significant depressive symptoms. Sleep latency was shorter on free-days compared to work-days among parents. Additionally, descriptive results suggested a trend toward increased sleep latency among fathers during the COVID-19 pandemic.

**Table 2:** Sleep characteristics of children, mothers and fathers by child age and COVID-19 pandemic periods.

	**Child age (years)**															
	**5**		**6**		**7**				**8**		**9**				**10**	
	**Pre-pandemic**		**Pre-pandemic**		**Pre-pandemic**		**Covid-19 pandemic**		**Covid-19 pandemic**		**Covid-19 pandemic**		**Post-pandemic**		**Post-pandemic**	
	***N***	**Mean (SD)**	***N***	**Mean (SD)**	***N***	**Mean (SD)**	***N***	**Mean (SD)**	***N***	**Mean (SD)**	***N***	**Mean (SD)**	***N***	**Mean (SD)**	***N***	**Mean (SD)**
**Child**																
**Sleep quality (CSHQ)**																
Boys	208	43.7 (5.5)	187	43.0 (6.0)	123	42.9 (6.7)	29	41.7 (5.7)	146	42.5 (6.2)	113	41.4 (4.8)	7	46.3 (7.2)	98	41.2 (4.8)
Girls	205	43.0 (5.8)	192	42.6 (5.5)	141	42.4 (5.7)	31	43.2 (4.7)	148	41.9 (5.3)	120	41.1 (5.7)	10	41.9 (3.7)	106	41.1 (5.0)
**Sleep duration (h)**																
**Week-days**																
Boys	247	10.6 (0.6)	202	10.5 (0.6)	129	10.3 (0.6)	29	10.3 (0.6)	152	10.0 (0.6)	126	9.8 (0.6)	7	9.5 (0.9)	112	9.6 (0.7)
Girls	240	10.6 (0.6)	207	10.4 (0.6)	150	10.2 (0.6)	30	10.3 (0.6)	160	10.0 (0.5)	125	9.7 (0.6)	9	10.0 (0.5)	117	9.5 (0.6)
**Free-days**																
Boys	206	10.7 (0.7)	183	10.5 (0.7)	125	10.5 (0.6)	29	10.3 (0.7)	137	10.2 (0.7)	128	9.9 (0.8)	7	10.1 (0.5)	92	9.7 (0.8)
Girls	200	10.8 (0.7)	185	10.7 (0.7)	146	10.5 (0.7)	30	10.6 (0.8)	140	10.3 (0.8)	123	10.1 (0.9)	9	10.6 (0.7)	100	10.1 (0.9)
**Sleep latency (min)**																
**Week-days**																
Boys	247	12.2 (8.5)	205	12.7 (9.9)	130	12.0 (9.5)	29	12.8 (9.5)	156	16.6 (16.4)	131	14.8 (12.0)	7	20.0 (19.8)	114	15.5 (14.5)
Girls	243	12.8 (8.8)	209	14.0 (10.6)	150	14.6 (13.1)	31	12.7 (8.0)	162	15.0 (11.1)	127	15.5 (10.7)	10	11.5 (7.8)	121	14.4 (10.1)
**Free-days**																
Boys	246	12.5 (9.1)	205	13.0 (11.9)	128	11.8 (9.9)	29	13.6 (9.9)	156	14.7 (12.9)	131	13.5 (11.8)	7	20.0 (19.8)	112	13.8 (14.9)
Girls	241	12.6 (8.8)	209	14.0 (11.3)	150	12.9 (10.8)	31	11.6 (8.5)	162	13.9 (11.9)	127	13.9 (10.0)	10	8.0 (4.2)	120	12.1 (9.2)
Work-days	–	–	368	4.6 (2.5)	261	4.7 (2.4)	52	4.8 (3.0)	302	4.4 (2.6)	236	4.6 (2.6)	12	5.2 (2.4)	–	–
Free-days	–	–	344	3.6 (2.2)	249	3.5 (2.2)	49	3.5 (2.2)	287	3.4 (2.4)	233	3.8 (2.4)	13	3.4 (2.3)	–	–
**Sleep duration (h)**																
Work-days	–	–	382	6.8 (0.8)	267	6.8 (0.9)	57	6.9 (1.2)	313	6.9 (0.9)	245	6.8 (0.9)	13	7.0 (0.9)	–	–
Free-days	–	–	389	7.6 (1.0)	272	7.7 (0.9)	58	8.0 (1.3)	316	7.7 (1.0)	249	7.8 (1.0)	14	7.6 (0.8)	–	–
**Time spent in bed (h)**																
Work-days	–	–	400	7.8 (0.8)	279	7.7 (0.8)	59	7.7 (0.8)	316	7.8 (0.8)	250	7.7 (0.8)	15	8.0 (0.8)	–	–
Free-days	–	–	401	8.4 (1.0)	279	8.5 (0.9)	59	8.6 (0.8)	318	8.5 (0.9)	250	8.5 (1.0)	15	8.2 (1.0)	–	–
**Sleep efficiency**																
Work-days	–	–	380	87.6 (9.7)	266	88.7 (9.7)	56	89.8 (13.1)	311	89.5 (10.5)	242	89.0 (9.6)	13	88.1 (8.8)	–	–
Free-days	–	–	381	90.9 (12.1)	271	90.8 (11.5)	56	91.3 (9.5)	314	91.0 (11.0)	245	92.3 (11.8)	14	93.8 (13.8)	–	–
**Sleep latency (min)**																
Work-days	–	–	398	11.7 (11.5)	278	12.0 (13.2)	58	11.5 (13.6)	314	12.2 (11.1)	249	11.2 (12.5)	15	13.5 (9.9)	–	–
Free-days	–	–	399	11.2 (12.7)	274	11.1 (11.2)	58	11.4 (11.4)	315	11.5 (11.5)	247	9.8 (8.7)	15	13.1 (10.0)	–	–
**Paternal**																
**Sleep quality (PSQI)**																
Work-days	–	–	265	4.6 (2.5)	184	4.5 (2.5)	38	4.6 (2.4)	206	4.4 (2.6)	161	4.3 (2.4)	11	4.9 (3.7)	–	–
Free-days	–	–	253	3.2 (2.2)	179	3.1 (2.1)	36	3.7 (2.7)	201	2.9 (2.1)	155	3.0 (2.4)	12	4.3 (3.3)	–	–
**Sleep duration (h)**																
Work-days	–	–	272	6.5 (0.9)	187	6.6 (0.8)	38	6.7 (0.9)	209	6.7 (0.8)	162	6.7 (0.9)	12	6.5 (1.0)	–	–
Free–days	–	–	267	7.5 (0.9)	187	7.5 (0.9)	39	7.6 (1.0)	210	7.6 (0.9)	163	7.7 (0.9)	12	7.5 (1.2)	–	–
**Time spent in bed (h)**																
Work–days	–	–	271	7.4 (0.9)	188	7.3 (0.9)	40	7.2 (0.8)	214	7.4 (0.9)	163	7.4 (0.9)	12	7.8 (1.2)	–	–
Free–days	–	–	267	8.1 (1.1)	188	8.1 (1.1)	40	8.2 (1.3)	212	8.1 (1.1)	162	8.2 (1.1)	12	8.9 (1.2)	–	–
**Sleep efficiency**																
Work–days	–	–	271	89.1 (10.4)	187	89.9 (9.7)	38	93.4 (10.7)	209	90.1 (11.0)	161	90.5 (8.5)	12	84.5 (14.4)	–	–
Free–days	–	–	262	93.0 (10.9)	186	92.8 (9.9)	39	93.3 (11.3)	209	94.9 (12.9)	161	93.3 (10.3)	12	85.3 (15.1)	–	–
**Sleep latency (min)**																
Work–days	–	–	271	9.7 (9.5)	187	9.1 (8.2)	40	14.6 (21.2)	213	9.9 (8.9)	163	10.6 (12.5)	12	13.1 (22.5)	–	–
Free–days	–	–	267	9.3 (9.1)	186	8.5 (7.0)	39	12.8 (20.2)	211	9.3 (9.3)	160	9.7 (10.4)	12	22.1 (50.4)	–	–

CSHQ, Children's Sleep Habits Questionnaire (higher scores indicate more sleep problems); PSQI, Pittsburgh Sleep Quality Index (higher score indicate more sleep problems); h, hours.

Tables 3, Supplementary Tables S1, S2 summarize the results of the Pearson correlation analyses. Among 6-year-old boys, a slight positive correlation was observed between their sleep quality and maternal sleep quality on both weekdays (*r* = 0.19, *p* = 0.01) and free-days (*r* = 0.22, *p* = 0.005), whereas no statistically significant correlation was found for girls (weekdays: *r* = 0.14, *p* = 0.06; free-days: *r* = 0.10, *p* = 0.19) (Table 3). At child age 8 years, no statistically significant correlations were observed for either boys or girls (Table 3). Stronger correlations were identified between maternal mental health and children's sleep quality, particularly among boys at ages 5, 6, and 9 years (*r* = 0.33, *p* < 0.001; *r* = 0.28, p <0.001; *r* = 0.25, *p* = 0.01) and among 5-year-old girls (*r* = 0.25, *p* < 0.001) (Supplementary Table S1). Maternal mental health was also strongly correlated with parental sleep quality, as detailed in Supplementary Table S2.

**Table 3:** Correlations between child sleep quality (by gender) and maternal and paternal sleep quality at child ages 6 and 8 years (pre–pandemic and during COVID−19 pandemic).

	**Maternal Sleep (PSQI)**				**Paternal Sleep (PSQI)**			
	**Weekday**		**Weekend**		**Weekday**		**Weekend**	
	***n***	**Pearson** ***r*** **(*****p–*****value)**	***n***	**Pearson** ***r*** **(*****p–*****value)**	***n***	**Pearson** ***r*** **(*****p–*****value)**	***n***	**Pearson** ***r*** **(*****p–*****value)**
**Child aged 6 (pre–pandemic)**								
**Child sleep (CSHQ)**								
Boys	171	0.19 (0.01)^*^	169	0.22 (0.005)^**^	123	0.06 (0.51)	120	−0.02 (0.82)
Girls	176	0.14 (0.06)	172	0.10 (0.19)	123	0.09 (0.32)	121	0.09 (0.31)
Maternal sleep (PSQI)	–	–	–	–	251	0.07 (0.30)	238	0.17 (0.009)^**^
**Child aged 8 (COVID**−**19 pandemic)**								
**Child sleep (CSHQ)**								
Boys	141	0.14 (0.11)	135	0.12 (0.18)	99	0.07 (0.47)	101	0.15 (0.13)
Girls	140	0.16 (0.06)	138	0.05 (0.55)	100	0.16 (0.11)	99	0.13 (0.21)
Maternal sleep (PSQI)					200	0.04 (0.61)	201	0.01 (0.85)

^*^p < 0.05; ^**^p < 0.01; Spearman correlation coefficients were almost identical to the reported Pearson correlation coefficients.

CSHQ, Children's Sleep Habits Questionnaire (higher scores indicate more sleep problems); PSQI, Pittsburgh Sleep Quality Index (higher scores indicate more sleep problems).

### Analytical results

Table 5 presents the results of the mixed models. During the COVID-19 pandemic, boys exhibited improved sleep quality compared to pre- and post-pandemic periods, with a mean intra-individual difference of −1.52 (95% confidence interval (CI): −2.85, −0.19). Additionally, daytime sleepiness decreased in both boys (−1.24; 95% CI: −1.84, −0.63) and girls (−1.52; 95% CI: −2.85, −0.19). However, no significant changes were observed in sleep latency or sleep duration among children during the COVID-19 pandemic. Among parents, mothers experience a slight increase in sleep duration on free-days (+0.26 h; 95% CI: 0.06, 0.46), while fathers reported increased sleep duration on work-days (+0.18 h; 95% CI: 0.01, 0.35). Fathers also showed an improvement in sleep efficiency (+4.81; 95% CI: 2.15, 7.46). Sleep latency and overall sleep quality, as measured by the PSQI remained largely unchanged for both mothers and fathers during the COVID-19 pandemic, regardless of work-days or free-days.

## Discussion

In this prospective study including data of 558 children, 465 mothers, and 318 fathers of the Ulm SPATZ Health study, we investigated the associations between the COVID-19 pandemic and various sleep characteristics. The analysis revealed that family sleep quality did not decline during the COVID-19 pandemic compared to pre- and post-pandemic periods. Notably, parental sleep duration increased, daytime sleepiness among children decreased, and boys experienced improved sleep quality during the COVID-19 pandemic.

### Sleep quality

Our finding of improved sleep quality among boys during the COVID-19 pandemic (CSHQ total score −1.52; 95 % CI: −2.85, −0.19) contrasts with most previous literature, which report a decline in children's sleep quality during this period (27, 32, 52, 53). However, many studies are cross-sectional or rely on retrospective assessment of pre-pandemic data, which may limit their comparability to our longitudinal approach. Two studies, however, have reported improved sleep quality during the COVID-19 pandemic. Genta et al. (54) examined 94 high school students attending a private Rudolf Steiner Waldorf High School in Brazil and found that sleep quality improved during the COVID-19 pandemic, particularly among those with shorter sleep duration before the COVID-19 pandemic. While this study did not assess the pre-pandemic data retrospectively, its small sample size and lack of representativeness for adolescents in Brazil are notable limitations. The relatively high socioeconomic status in both Genta et al.'s sample and our cohort may partially explain the observed improvements. Similarly, Liu et al. (20) analyzed data from 1,619 Chinese preschoolers aged 4–6 years and reported higher sleep quality during the COVID-19 pandemic compared to a socio-demographically similar group of 436 preschoolers assessed in 2018. However, this study involved younger children than those in our cohort, and a key limitation was the lack of adjustment for potential confounders, which may have introduced bias. Together, these findings suggest that improved sleep quality in children during a pandemic period may be influenced by factors such as socioeconomic status and age. These results highlight the importance of clearly defining the study population, as certain demographic characteristics may play a crucial role in shaping sleep outcomes.

Our findings did not suggest substantial changes in sleep quality among parents during the COVID-19 pandemic (Table 4). In contrast, Olive et al. (30) reported decreased sleep quality in 2,365 Australian parents, while a systematic review and meta-analysis by Jahrami et al. (31) found an increased prevalence of sleep problems among adults globally during the COVID-19 pandemic, with prevalence rates varying by country. Notably, the prevalence in Germany aligned with the global prevalence ratio of ~35%. Similarly, Cui et al. (55) identified decreased sleep quality and increased sleep duration among adults in their meta-analysis, findings that were echoed by Alimoradi et al. (56) Silva et al. (32) further observed an association between parental and child sleep problems during the COVID-19 pandemic, though we did not adjust for child sleep problems in our analysis due to the unclear direction of this relationship. Kocevska et al. (57) highlighted the importance of pre-pandemic sleep patterns, noting an unexpected trend: individuals with pre-existing insomnia showed improved sleep quality during the COVID-19 pandemic, while those with good pre-pandemic sleep experienced a decline. Unlike many studies relying on retrospective pre-pandemic data or cross-sectional designs, our analysis incorporated longitudinal data using mixed models and up to four follow-up measurement points, which accounted for pre-pandemic sleep quality. This approach reduces potential biases and strengthens the causal interpretation of our findings. While numerous studies have reported adverse changes in adult sleep during the COVID-19 pandemic, methodological differences likely contribute to the variation in results. For example, Leone et al. (11) using a longitudinal study design, found no changes in sleep quality among adults (*n* = 1021) during the COVID-19 pandemic, consistent with our findings. Of note, our study examined the entire pandemic period in Germany, spanning ~2 years, whereas many studies focused specifically on the early confinement measures implemented in March 2020. This broader time frame may also explain the difference in findings.

**Table 4:** Mean differences and 95% confidence intervals (CI) for sleep characteristics in children (by gender) and parents during the COVID−19 pandemic compared to pre– and post–pandemic periods.

	**Basic model**			**Full model**		
**Sleep characteristics**	**Subjects/ observations**	**Mean difference(95% CI)**	***P*** **–value**	**Subjects/ observations**	**Mean difference**^4, 5^ **(95% CI)**	***P*** **–value**
**Child sleep** ^1^						
**Sleep quality (CSHQ total score)**						
Boys	278/897	−1.39 (−2.64, −0.13)	0.03^*^	243/859	−1.52 (−2.85, −0.19)	0.03^*^
Girls	280/953	−0.75 (−1.86, 0.35)	0.17	245/881	−0.76 (−1.98, 0.46)	0.22
**subscales**						
**Daytime sleepiness (CSHQ**−**8)**						
Boys	278/982	−1.18 (−1.76, −0.61)	<0.001^**^	243/935	−1.24 (−1.84, −0.63)	<0.001^**^
Girls	280/1,030	−0.38 (−0.96, 0.21)	0.21	245/955	−0.73 (−1.34, −0.11)	0.02^*^
**Sleep duration week–days (h)**						
Boys	278/992	+0.01 (−0.14, 0.17)	0.91	243/944	+0.02 (−0.14, 0.19)	0.78
Girls	280/1,038	+0.13 (−0.02, 0.28)	0.10	245/965	+0.08 (−0.10, 0.25)	0.38
**Sleep duration free–days (h)**						
Boys	278/981	−0.13 (−0.33, 0.07)	0.19	243/936	−0.13 (−0.34, 0.07)	0.21
Girls	280/1,023	0.09 (−0.11, 0.28)	0.37	245/951	0.01 (−0.20, 0.23)	0.90
**Sleep latency week–days (min)**						
Boys	278/1,005	−0.76 (−3.44, 1.92)	0.58	243/954	−1.83 (−4.58, 0.91)	0.19
Girls	280/1,053	−0.06 (−3.14, 3.01)	0.97	245/979	0.23 (−3.28, 3.74)	0.90
**Sleep latency free–days (min)**						
Boys	278/1,000	0.55 (−2.11, 3.22)	0.68	243/950	−1.08 (−3.79, 1.63)	0.43
Girls	280/1,050	1.10 (−1.68, 3.87)	0.44	245/976	1.04 (−2.08, 4.17)	0.51
**Maternal sleep** ^2^						
**Work–days**						
Maternal sleep quality (PSQI)	465/1,226	−0.17 (−0.65, 0.31)	0.49	403/1,137	−0.21 (−0.74, 0.32)	0.43
Maternal sleep duration	465/1,271	+0.10 (−0.07, 0.27)	0.26	403/1,180	+0.09 (−0.10, 0.28)	0.35
Maternal time spent in bed	465/1,312	−0.02 (−0.15, 0.12)	0.80	403/1,219	+0.02 (−0.17, 0.12)	0.75
Maternal sleep efficiency	465/1,262	+1.79 (−0.35, 3.94**)**	0.10	403/1,172	+2.05 (−0.32, 4.42)	0.10
Maternal sleep latency	465/1,305	−0.61 (−2.87, 1.65)	0.60	403/1,212	−0.58 (−3.05, 1.90)	0.65
**Free–days**						
Maternal sleep quality (PSQI)	465/1,210	+0.12 (−0.32, 0.56)	0.09	403/1,127	+0.08 (−0.40, 0.56)	0.75
Maternal sleep duration	465/1,291	+0.26 (0.06, 0.46)	0.01^*^	403/1,199	+0.31 (0.09, 0.53)	0.007^**^
Maternal time spent in bed	465/1,315	+0.16 (−0.02, 0.33)	0.07	403/1,221	+0.12 (−0.07, 0.31)	0.22
Maternal sleep efficiency	465/1,274	−0.27 (−2.81, 2.26)	0.83	403/1,182	0.66 (−2–14, 3.46)	0.64
Maternal sleep latency	465/1,301	−0.20 (−2.07, 1.68)	0.84	403/1,209	0.27 (−1.79, 2.32)	0.80
**Sleep characteristics**	**Subjects/ observations**	**Mean difference(95% CI)**	***P*** **–value**	**Subjects/ observations**	**Mean difference**^4, 5^ **(95% CI)**	***P*** **–value**
**Paternal sleep** ^3^						
**Work–days**						
Paternal sleep quality (PSQI)	318/857	−0.39 (−91, 0.14)	0.15	267/787	−0.31 (−0.88, 0.26)	0.28
Paternal sleep duration	318/872	+0.18 (0.01, 0.35)	0.04^*^	267/802	+0.16 (−0.03, 0.35)	0.10
Paternal time spent in bed	318/880	−0.12 (−0.29, 0.05)	0.18	267/808	−0.15 (−0.34, 0.04)	0.11
Paternal sleep efficiency	318/870	+4.50 (2.14, 6.87)	<0.001^**^	267/800	+4.81 (2.15, 7.46)	<0.001^**^
Paternal sleep latency	318/878	1.34 (−0.94, 3.62)	0.25	267/806	1.65 (−0.80, 4.11)	0.19
**Free–days**						
Paternal sleep quality (PSQI)	318/840	−0.22 (−0.72, 0.27)	0.38	267/771	−0.12 (−0.67, 0.43)	0.66
Paternal sleep duration	318/870	+0.09 (−0.11, 0.28)	0.39	267/799	+0.10 (−0.11, 0.32)	0.35
Paternal time spent in bed	318/873	−0.13 (−0.36, 0.10)	0.27	267/802	−0.13 (−0.39, 0.12)	0.31
Paternal sleep efficiency	318/861	+2.45 (−0.38, 5.28)	0.09	267/791	+2.35 (−0.80, 5.50)	0.14
Paternal sleep latency	318/867	−0.89 (−2.98, 1.21)	0.41	267/797	−1.22 (−3.49, 1.04)	0.29

^*^p < 0.05; ^**^p < 0.01.

^1^Models were adjusted for child age at the time of the assessment, child age at school entry, maternal educational attainment (<12 years or ≥12 years of school education), maternal nationality (German or non–German), and maternal age at childbirth.

^2^Models were adjusted for child age at the time of the assessment, maternal age at childbirth, maternal educational attainment (<12 years or ≥12 years of school education), and maternal nationality (German or non–German).

^3^Models were adjusted for child age at the time of the assessment, paternal age at childbirth, paternal educational attainment (<12 years or ≥12 years of school education), and paternal nationality (German or non–German).

^4^Adjustement as in 1/2 and additionally adjusted for maternal mental health at start of the respective outcome modeling (models for child and maternal sleep characteristics).

^5^Adjustement as in 3 and additionally adjusted for paternal mental health at start of the respective outcome modeling (models for paternal sleep characteristics).

Child: Six waves of the SPATZ study were used (child age 5–10 years), because ‘wetting during night' is included in the CSHQ from 5 years onwards. Parents: Four waves of the SPATZ study were used (child age 6–9 years), because parents–sleep was assessed with same instrument during this time window (PSQI differentiated in work–days and free–days).

CSHQ , Children's Sleep Habits Questionnaire (higher scores indicate more sleep problems); PSQI , Pittsburgh Sleep Quality Index (higher scores indicate more sleep problems).

The distribution of adjustment variables is shown in Table 1 and Supplementary Table S3.

### Sleep duration

Our analysis indicated that sleep duration among children did not increase during the COVID-19 pandemic (Table 4). Similarly, Luchini et al. (58) reported no increase in sleep duration in their study of 528 children aged 4–12 years in the United States during the COVID-19 pandemic. However, Poirier et al. (18) found that among 28,307 Canadian adolescents, average sleep duration increased by 9.6 min between 2019 and 2022, alongside an increase in screen time by 129.2 min (95% CI: 120.5, 138.0). The differing age ranges of the study population may partly explain these contrasting findings. Additionally, while numerous studies have reported increased sleep duration among children and adolescents during the COVID-19 pandemic, (2, 4–10) our results did not align with this observation.

A multi-center study conducted in Bavaria, Germany, including 1,962 children (63.4% in primary schools and 36.6% in daycare centers), observed that children who experienced changes in sleep duration during the COVID-19 pandemic—whether an increase or decrease—reported worse health-related quality of life compared to those whose sleep duration remained stable (59). This finding aligns with numerous studies that have reported increased sleep duration during the COVID-19 pandemic accompanied by decreased sleep quality, which is often linked to mental health challenges. These observations are partially consistent with our findings among boys, where sleep duration remained unchanged, but sleep quality improved during the COVID-19 pandemic (Table 4).

We observed increased sleep duration among parents during the COVID-19 pandemic, specifically for mothers on weekends (difference in means +0.31 h; 95% CI 0.09, 0.53) and fathers on weekdays (difference in means +0.18 h; 95% CI 0.01, 0.35). These findings are consistent with numerous studies that have reported an increase in sleep duration among adults during the COVID-19 pandemic (11, 21, 60–62). However, results for adults remain mixed, as some studies found no change in sleep duration, (63, 64) while others reported divergent trends depending on work conditions—sleep duration increased with remote work and decreased for those continuing to work in person (65). Many studies, including ours, did not differentiate between these work settings. A further limitation of adult sleep studies is that large datasets often use time in bed as a proxy for actual sleep duration, which can lead to inaccuracies (61). For example, Hisler and Twenge (64) found no change in average sleep duration in a nationally representative sample of U.S. adults when comparing 2020 to 2018. However, they noted an increase in the prevalence of both shorter and longer sleep durations outside recommended ranges during the COVID-19 pandemic. Our analysis focused on intra-individual changes and predicted the mean of these changes, adjusted for covariates. While we identified increases in sleep duration among mothers on weekends and fathers on weekdays, our results do not exclude the possibility that these increases contributed to a higher prevalence of sleep durations falling outside the recommended ranges.

### Daytime sleepiness

The reduction in daytime sleepiness observed among boys in this study may be linked to the delayed school start times during the COVID-19 pandemic, as later school start times have been shown to improve daytime sleepiness (66). This effect was also reported during the COVID-19 pandemic in a study of 45 Canadian adolescents (67).

Moreover, research has shown that daytime sleepiness is negatively associated with health related quality of life in schoolchildren (68). Our findings align with these observations to some extent. While boys experienced a significant reduction in daytime sleepiness during the COVID-19 pandemic, their health-related quality of life remained unchanged, as observed in a previous study of the same cohort (12, 17).

The results of our study, in conjunction with the research conducted by Maciel et al. (69) suggest that the implementation of flexible school start times may contribute to the enhancement of sleep characteristics in children and adolescents.

### Correlations between sleep qualities in families

We did not observe a substantial correlation between maternal or paternal sleep quality and child sleep quality during the COVID-19 pandemic (Table 3). This contrasts with findings from Zreik et al. (70) who reported a strong association between maternal and child sleep. Specifically, mothers who reported improved sleep quality or longer sleep duration in their children during the COVID-19 pandemic were also more likely to experience a reduction in their own sleep problems. These findings are further supported by Tikotzky et al. (71) who demonstrated that maternal sleep is predictive of infant sleep during the first 6 months of life under non-pandemic conditions. As Zreik et al. studied children aged 0.5 to 6 years, and we found only a weak correlation between maternal and child sleep quality for 6 year old boys—an association that was no longer present when boys reached 8 years—it is possible that the relationship between maternal and child sleep diminishes as children grow older.

### Mental health and sleep quality

In two earlier studies using the same cohort, we found that girls, but not boys, experienced poorer mental health during the COVID-19 pandemic compared to before (12, 17). This finding aligns with our observation of improved sleep quality among boys during the COVID-19 pandemic, suggesting that better sleep quality may have contributed to maintaining mental health in boys during this period. Improved sleep quality is causally linked to better mental health in adults (72), while increasing sleep disturbances are well-established risk factors for mental health issues (14). Conversely, poor mental health can also negatively affect sleep quality (45), highlighting the bidirectional and complex relationship between sleep and mental health, which may be further complicated during a pandemic (4, 21). In our cohort, the absence of poorer sleep quality among girls, despite their lower mental health, supports the hypothesis that sleep quality likely contributed to good mental health during the COVID-19 pandemic rather than being a consequence of it. If poor mental health were the primary driver of sleep disturbances, we would expect girls to exhibit worse sleep quality in this analysis.

We also identified a correlation between maternal mental health and boys' sleep quality at ages 5, 6 and 9 years, but this relationship was observed among girls at age 5 years (Supplementary Table S1). This suggests a possible gender difference in the associations between parental mental health and child sleep quality, although the direction of this association remains unclear. Additionally, we found strong correlations between parental sleep quality and their own mental health status (Supplementary Table S2), consistent with the bidirectional relationship between sleep and mental health (4, 21).

#### Additional adjustment for mental health in the analysis

Adjusting for pre-pandemic mental health did not substantially alter the associations between the COVID-19 pandemic and sleep characteristics, except for paternal sleep duration and girl's daytime sleepiness (Table 4).

After adjusting for the pre-pandemic HADS score (a summary score for mental health), the increase in sleep duration among fathers during the COVID-19 pandemic was no longer significant (Table 4). This suggests an inverse relationship between pre-pandemic HADS scores and sleep duration, as the model indicated that poorer pre-pandemic mental health (higher HADS scores) was associated with shorter sleep duration (data not shown). To explore this further, we stratified fathers based on their pre-pandemic HADS scores (<median vs. ≥ median, with a median HADS score of 7). Stratified analysis revealed that only fathers with good pre-pandemic mental health (HADS <7) experienced an increase in sleep duration during the COVID-19 pandemic (mean difference +0.22 h; 95% CI: 0.05, 0.40; *p* = 0.01), whereas fathers with HADS ≥ 7 showed no significant change (mean difference +0.13 h; 95% CI −0.15, 0.40; *p* = 0.36).

We observed reduced daytime sleepiness among girls during the COVID-19 pandemic, but only after adjusting for maternal pre-pandemic mental health. This adjustment likely reduced variability in the model and accounted for the positive association between maternal HADS scores and girls' daytime sleepiness observed in the analysis (data not shown). As such, the findings of reduced daytime sleepiness in girls' should be interpreted carefully, as they represent an association independent of maternal pre-pandemic mental health, and effect modification between pre-pandemic mental health and the COVID-19 pandemic cannot be ruled out.

### Limitations

Our findings should be interpreted with caution given the study's limitations. First, the relatively small sample size limits statistical power and may affect the robustness of some associations. Second, our sample had a high proportion of families with mothers who had higher educational attainment at baseline. While this reflects the local population around a university city, families with lower educational attainment or migrant backgrounds were more likely to be lost to follow-up, particularly during the first year. Third, children's sleep quality was assessed using parent-proxy reports rather than self-reports. However, given that we adjusted for maternal mental health and found little to no association between parental sleep quality and child sleep quality, the use of a parent-proxy is unlikely to have introduced substantial bias, and the findings on children's sleep characteristics can be considered reliable. However, even though the CSHQ and PSQI are validated tools for assessing sleep quality, relying on self/parent-reported data may have introduced recall bias, affecting the accuracy of our findings. Moreover, our study did not include measurements of sleep characteristics by means of actigraphy, which could have introduced bias as we only relied on self-reported data on sleep characteristics. Further studies may validate reported sleep characteristics with a subsample of their study population using more objective measurements, such as actigraphy or daily sleep protocols. Another limitation is that our statistical model assumes no age-specific differences in the association between the pandemic and children's sleep characteristics across ages 7, 8 and 9 years. Effect modification by age was not tested, which could limit insights into potential age-related variability in pandemic effects.

Since our analysis treated the pandemic as a single event spanning 2 years, future research might consider multiple phases, such as the initial lockdown, phased reopening and vaccine rollout, if sample sizes allow. This would facilitate a deeper understanding of the pandemic and its associations with sleep health. Further, as our sample size was too small to divide the time before and after the pandemic into 'before' and 'after' time windows and compare these time windows separately with the time during the pandemic, our analysis is limited by this. Further research should aim to obtain more post-pandemic measurements in order to specifically explore the period after the pandemic compared to the period during the pandemic.

Moreover, as we considered the CHSQ and PSQI on a linear scale to explore potential associations between the COVID-19 pandemic and sleep quality, further research should also consider meaningful changes and clinically relevant thresholds for sleep quality.

Another limitation is that our analysis did not consider whether parents were working remotely or in person during the pandemic, even though this can influence changes to sleep duration during a pandemic.

The key strength of our study lies in its longitudinal design, which enabled the collection of routinely gathered data even during the unpredictable conditions of the COVID-19 pandemic. This design enhances the reliability of the findings and provides a valuable record of the possible associations of the pandemic with health of families and children in a natural experiment, as captured by the Ulm SPATZ Health study (73). As our analysis was exploratory rather than confirmatory, the results should be interpreted with caution. Further research involving specific hypothesis testing is necessary to gain a deeper understanding of the pandemic-related impact on sleep health.

## Conclusion

Despite these limitations, our findings indicated that sleep quality of families did not generally decline during the COVID-19 pandemic compared to before and after the COVID-19 pandemic. However, we observed an increase in parental sleep duration, a decrease in daytime sleepiness among children, and an improvement in sleep quality among boys during this time. Further studies are required to assess the long-term implications of the pandemic on sleep characteristics, particularly among vulnerable groups or individuals with pre-existing mental health conditions.

## Data Availability

The datasets presented in this article are not readily available because the datasets generated during and/or analyzed during the current study are not publicly available due to ethical restrictions regarding data protection issues and the study-specific consent text and procedure, but anonymized data are available from the corresponding author upon reasonable request. Supplementary results are available upon request. Requests to access the datasets should be directed to deborah.wernecke@uni-ulm.de.
