# Supplementary material for: Longitudinal associations between the COVID-19 pandemic and sleep characteristics in children and parents
Source: Front Public Health. 2025 Sep 25;13:1604195. doi: 10.3389/fpubh.2025.1604195 (PMC12507763; doi:10.3389/fpubh.2025.1604195)
Supplement: Supplementary file 1 [file Data_Sheet_1.docx]

**Table S1.** Correlations between child sleep quality (CSHQ) and maternal mental health (HADS) at child ages 5, 6, and 9 years.

|  | Maternal Mental Health (HADS) | | | | | |
| --- | --- | --- | --- | --- | --- | --- |
|  | Child age 5 years | | Child age 6 years | | Child age 9 years | |
|  | n | Pearson r  (p-value) | n | Pearson r  (p-value) | n | Pearson r  (p-value) |
| Child sleep (CSHQ) |  |  |  |  |  |  |
| Boys | 208 | 0.33 (<0.001)** | 186 | 0.28 (<0.001)** | 104 | 0.25 (0.01)* |
| Girls | 203 | 0.25 (<0.001)** | 191 | 0.13 (0.07) | 115 | 0.09 (0.34) |

*p < 0.05; **p < 0.01; Spearman correlation coefficients were almost identical to the reported Pearson correlation coefficients.

CSHQ - Children's Sleep Habits Questionnaire; HADS - Hospital Anxiety and Depression Scale

**Table S2.** Correlations between parental sleep quality (PSQI) and mental health (HADS) at child ages 6 and 9 years.

|  | Maternal/paternal mental health at the respective time point (HADS) | | | |
| --- | --- | --- | --- | --- |
|  | Child age 6 years | | Child age 9 years | |
|  | n | Pearson r  (p-value) | n | Pearson r  (p-value) |
| Maternal sleep quality (PSQI) |  |  |  |  |
| Work-days | 380 | 0.40 (<0.0001)** | 228 | 0.38 (<0.0001)** |
| Free-days | 373 | 0.40 (<0.0001)** | 226 | 0.42 (<0.0001)** |
| Paternal sleep quality (PSQI) |  |  |  |  |
| Work-days | 270 | 0.49 (<0.0001)** | 161 | 0.61 (<0.0001)** |
| Free-days | 262 | 0.41 (<0.0001)** | 156 | 0.47 (<0.0001)** |

*p < 0.05; **p < 0.01; Spearman correlation coefficients were almost identical to the reported Pearson correlation coefficients.

: PSQI - Pittsburgh Sleep Quality Index; HADS - Hospital Anxiety and Depression Scale

Only data from child ages 6 and 9 years were used, as the PSQI was assessed separately for work-days and free-days at these time points, and HADS data were also available. At child age 5 years, data on both HADS and PSQI were available, but the PSQI was assessed for weekdays and weekends combined, preventing its inclusion in this analysis.

**Table S3.** Distribution of adjustement variables included in the multivariable mixed models.

|  | Analysis of child sleep | | Analysis of maternal sleep | | Analysis of paternal sleep | |
| --- | --- | --- | --- | --- | --- | --- |
|  | n | Mean (SD),  Median (IQR) | n | Mean (SD),  Median (IQR) | n | Mean (SD),  Median (IQR) |
| Child age at school entry (years) | 558 | 6.45 (0.28)  6.41 (6.22-6.68) | - | - | - | - |
| Maternal pre-pandemic HADS-score (child age 5 years) | 475 | 8.68 (5.57)  8.00 (5.00-12.00) | - | - | - | - |
| Maternal pre-pandemic HADS-score (child age 6 years) | - | - | 406 | 9.43 (5.70)  9.00 (5.00-13.00) | - | - |
| Paternal pre-pandemic HADS-score (child age 6 years) | - | - | - | - | 270 | 8.37 (5.62)  7.00 (4.00-12.00) |

IQR=interquartile range
